# EslB Is Required for Cell Wall Biosynthesis and Modification in Listeria monocytogenes

**DOI:** 10.1128/JB.00553-20

**Published:** 2021-01-25

**Authors:** Jeanine Rismondo, Lisa M. Schulz, Maria Yacoub, Ashima Wadhawan, Michael Hoppert, Marc S. Dionne, Angelika Gründling

**Affiliations:** a Section of Molecular Microbiology and Medical Research Council Centre for Molecular Bacteriology and Infection, Imperial College London, London, United Kingdom; b Department of General Microbiology, GZMB, Georg August University Göttingen, Göttingen, Germany; c Department of Life Sciences, Medical Research Council Centre for Molecular Bacteriology and Infection, Imperial College London, London, United Kingdom; Université de Montréal

**Keywords:** ABC transporters, lysozyme, cell division, cell wall

## Abstract

Lysozyme is an important component of the innate immune system. It functions by hydrolyzing the peptidoglycan (PG) layer of bacteria. The human pathogen Listeria monocytogenes is intrinsically lysozyme resistant. The peptidoglycan *N*-deacetylase PgdA and *O*-acetyltransferase OatA are two known factors contributing to its lysozyme resistance. Furthermore, it was shown that the absence of components of an ABC transporter, referred to here as EslABC, leads to reduced lysozyme resistance. How its activity is linked to lysozyme resistance is still unknown. To investigate this further, a strain with a deletion in *eslB*, coding for a membrane component of the ABC transporter, was constructed in L. monocytogenes strain 10403S. The *eslB* mutant showed a 40-fold reduction in the MIC to lysozyme. Analysis of the PG structure revealed that the *eslB* mutant produced PG with reduced levels of *O*-acetylation. Using growth and autolysis assays, we showed that the absence of EslB manifests in a growth defect in media containing high concentrations of sugars and increased endogenous cell lysis. A thinner PG layer produced by the *eslB* mutant under these growth conditions might explain these phenotypes. Furthermore, the *eslB* mutant had a noticeable cell division defect and formed elongated cells. Microscopy analysis revealed that an early cell division protein still localized in the *eslB* mutant, indicating that a downstream process is perturbed. Based on our results, we hypothesize that EslB affects the biosynthesis and modification of the cell wall in L. monocytogenes and is thus important for the maintenance of cell wall integrity.

**IMPORTANCE** The ABC transporter EslABC is associated with the intrinsic lysozyme resistance of Listeria monocytogenes. However, the exact role of the transporter in this process and in the physiology of L. monocytogenes is unknown. Using different assays to characterize an *eslB* deletion strain, we found that the absence of EslB affects not only lysozyme resistance but also endogenous cell lysis, cell wall biosynthesis, cell division, and the ability of the bacterium to grow in media containing high concentrations of sugars. Our results indicate that EslB is, by means of a yet-unknown mechanism, an important determinant for cell wall integrity in L. monocytogenes.

## Abstract (precis)

The ABC transporter EslABC is associated with the intrinsic lysozyme resistance of Listeria monocytogenes. However, the exact role of the transporter in this process and in the physiology of L. monocytogenes is unknown.

## INTRODUCTION

Gram-positive bacteria are surrounded by a complex cell wall, which is composed of a thick layer of peptidoglycan (PG) and cell wall polymers. The bacterial cell wall is important for the maintenance of cell shape, for the ability of bacteria to withstand harsh environmental conditions, and for preventing cell lysis (1, 2). Due to its importance, cell wall-targeting antibiotics such as β-lactam, glycopeptide, and fosfomycin antibiotics are commonly used to treat bacterial infections (3, 4). These cell wall-targeting antibiotics inhibit enzymes involved in different stages of the PG biosynthesis process or sequester substrates of these enzymes (4). Moenomycin, another cell wall-targeting antibiotic, and β-lactam antibiotics, for instance, block the glycosyltransferase and transpeptidase activities of penicillin-binding proteins, respectively, which are required for the polymerization and cross-linking of the glycan strands (5–7). Peptidoglycan is also the target of the cell wall hydrolase lysozyme, which is a component of animal and human secretions such as tears and mucus. Lysozyme cleaves the glycan strands of PG by hydrolyzing the 1,4-β-linkage between *N*-acetylmuramic acid (MurNAc) and *N*-acetylglucosamine (GlcNAc). This reaction leads to a loss of cell integrity and results in cell lysis (8).

**FIG 1:**
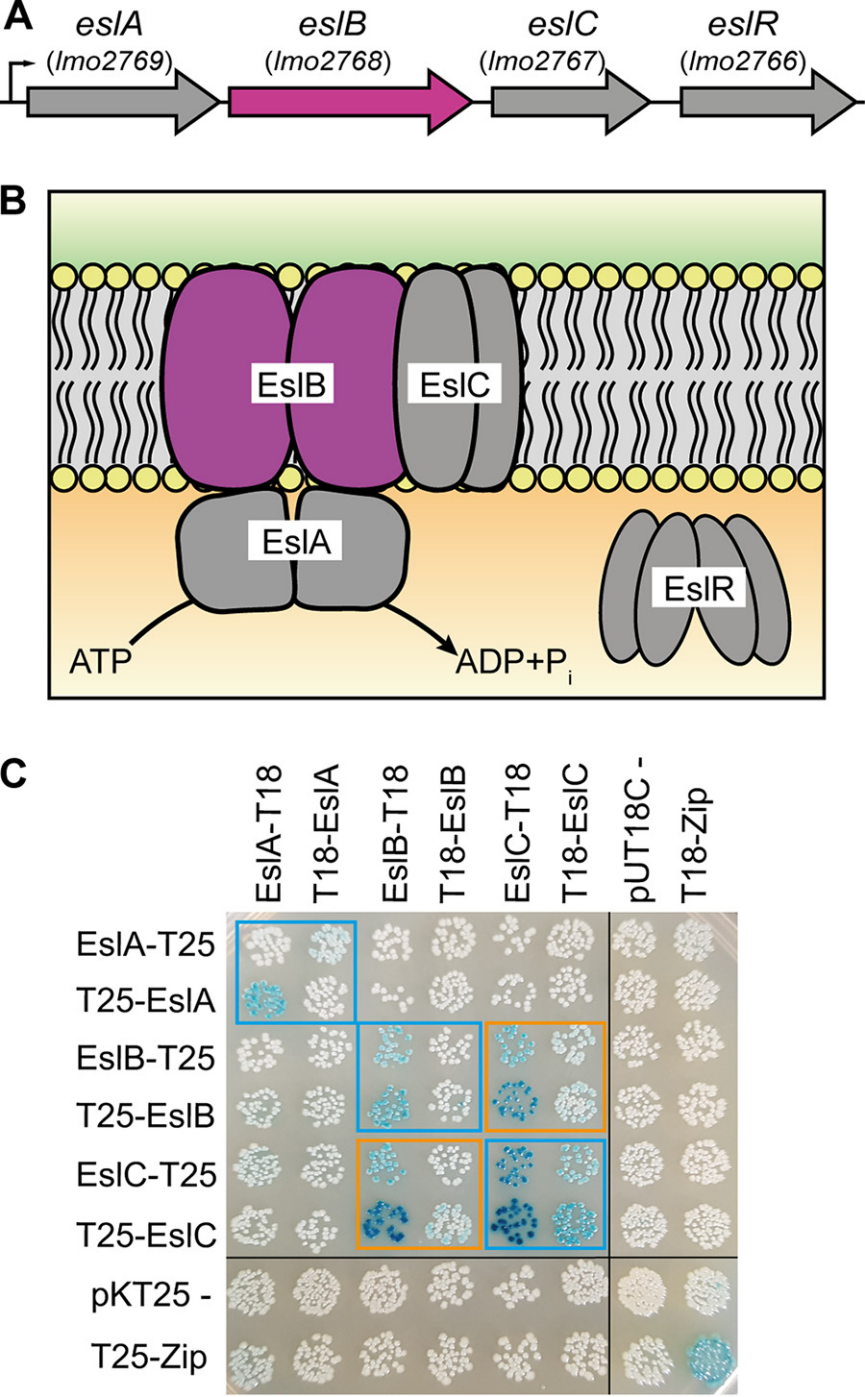
Schematic representation of the L. monocytogenes *eslABCR* operon and interaction of the ABC transporter components EslABC. (A) Genomic arrangement of the *eslABCR* operon in L. monocytogenes. Arrows indicate the orientation of the genes. The small black arrow indicates the promoter identified in a previous study (20). (B) Model of the ABC transporter composed of the NBD protein EslA, which hydrolyses ATP, the TMD proteins EslB and EslC, and the cytoplasmic RpiR family transcription regulator EslR. The *eslB* gene (A) and EslB protein (B), which were investigated as part of the study, are in pink. (C) Interactions between the ABC transporter components. Plasmids encoding fusions of EslA, EslB, and EslC and the T18 and T25 fragments of the Bordetella pertussis adenylate cyclase were cotransformed into E. coli BTH101. Empty vectors pKT25 and pUT18C were used as negative controls, and pKT25-Zip and pUT18C-Zip were used as positive controls. Black lines indicate where lanes which were not required were removed. Self-interactions are marked with blue boxes and protein-protein interactions are marked with orange boxes. A representative image of three repeats is shown.

The intracellular human pathogen Listeria monocytogenes is intrinsically resistant to lysozyme due to modifications of its PG. The *N*-deacetylase PgdA deacetylates GlcNAc residues, whereas MurNAc residues are acetylated by the *O*-acetyltransferase OatA (9, 10). Consequently, deletion of either of these enzymes results in reduced lysozyme resistance (9, 10). One or both of these enzymes are also present in other bacterial pathogens and important for lysozyme resistance, such as PgdA in Streptococcus pneumoniae, OatA in Staphylococcus aureus, and PgdA and OatA in Enterococcus faecalis (11–14). Besides enzymes that directly alter the peptidoglycan structure, a number of other factors have been shown to contribute to lysozyme resistance in diverse bacteria. For instance, the cell wall polymer wall teichoic acid and the two-component system GraRS contribute to lysozyme resistance in S. aureus (15, 16). In E. faecalis, the extracytoplasmic-function sigma factor SigV is required for the upregulation of *pgdA* expression in the presence of lysozyme (11, 17). Recently, some additional factors which contribute to the intrinsic lysozyme resistance of L. monocytogenes were identified, such as the predicted carboxypeptidase PbpX, the transcription factor DegU, and the noncoding RNA Rli31 (18). DegU and Rli31 are involved in the regulation of *pgdA* and *pbpX* expression in L. monocytogenes (18). Furthermore, components of a predicted ABC transporter encoded by the *lmo2769-6* operon in L. monocytogenes and referred to here as *eslABCR* (for “elongation and sugar- and lysozyme-sensitive phenotype”) (Fig. 1) have been associated with lysozyme resistance (18–20). An *eslB* transposon insertion mutant was also shown to be more sensitive to cefuroxime and cationic antimicrobial peptides (18).

ABC transporters can either act as importers or exporters. Importers are involved in the uptake of sugars, peptides, or other metabolites, which are recognized by substrate-binding proteins. On the other hand, toxic compounds such as antibiotics can be exported by ABC exporters (21–23). They are usually composed of homo- or heterodimeric cytoplasmic nucleotide-binding domain (NBD) proteins, also referred to as ATP-binding cassette proteins, and homo- or heterodimeric transmembrane domain (TMD) proteins (24). In addition to NBDs and TMDs, ABC importers have an extracellular substrate binding protein (SBP) or a membrane-integrated S component, which are important for the delivery of specific substrate molecules to the transporter or substrate binding, respectively (25–27).

The *esl* operon encodes EslA, the NBD protein, EslB, the TMD protein forming part of the ABC transporter, EslC, a membrane protein of unknown function, and EslR, an RpiR-type transcriptional regulator (Fig. 1). So far, it has not been investigated whether EslC is a component of the ABC transporter encoded in the *esl* operon. EslB and EslC could for instance interact with each other and form the transmembrane domain of the ABC transporter, or EslC could function independently of EslAB. Furthermore, it is not known whether the predicted ABC transporter EslABC acts as an importer or exporter, and its exact cellular function has not been identified. Here, we show that the absence of EslB, one of the transmembrane components of the ABC transporter, leads to an increased lysozyme sensitivity due to an altered PG structure. In addition, deletion of *eslB* resulted in the production of a thinner cell wall and thus to increased endogenous cell lysis. Furthermore, cell division is perturbed in the absence of EslB. We hypothesize that EslB may be required for processes which are important for the maintenance of the cell wall integrity of L. monocytogenes under stress conditions.

## RESULTS

### EslC interacts with the transmembrane protein EslB.

It was shown previously that L. monocytogenes strains with mutations in the *eslABCR* operon (Fig. 1A) display decreased resistance to the cell wall hydrolase lysozyme (18, 19). The *esl* operon encodes the ATP-binding protein EslA and the transmembrane proteins EslB and EslC, which are proposed to form an ABC transporter. However, it is currently unknown if EslC forms part of the ABC transporter as depicted in Fig. 1B and if it is required for the function of the transporter. To gain insights into the composition of the ABC transporter, we assessed the interaction between EslA, EslB, and EslC using the bacterial adenylate cyclase-based two-hybrid system. In addition to self-interactions of EslA, EslB, and EslC, we observed an interaction between EslB and EslC (Fig. 1C), indicating that EslC might be part of the ABC transporter.

### Deletion of *eslB* in L. monocytogenes leads to lysozyme sensitivity and an altered peptidoglycan structure.

**FIG 2:**
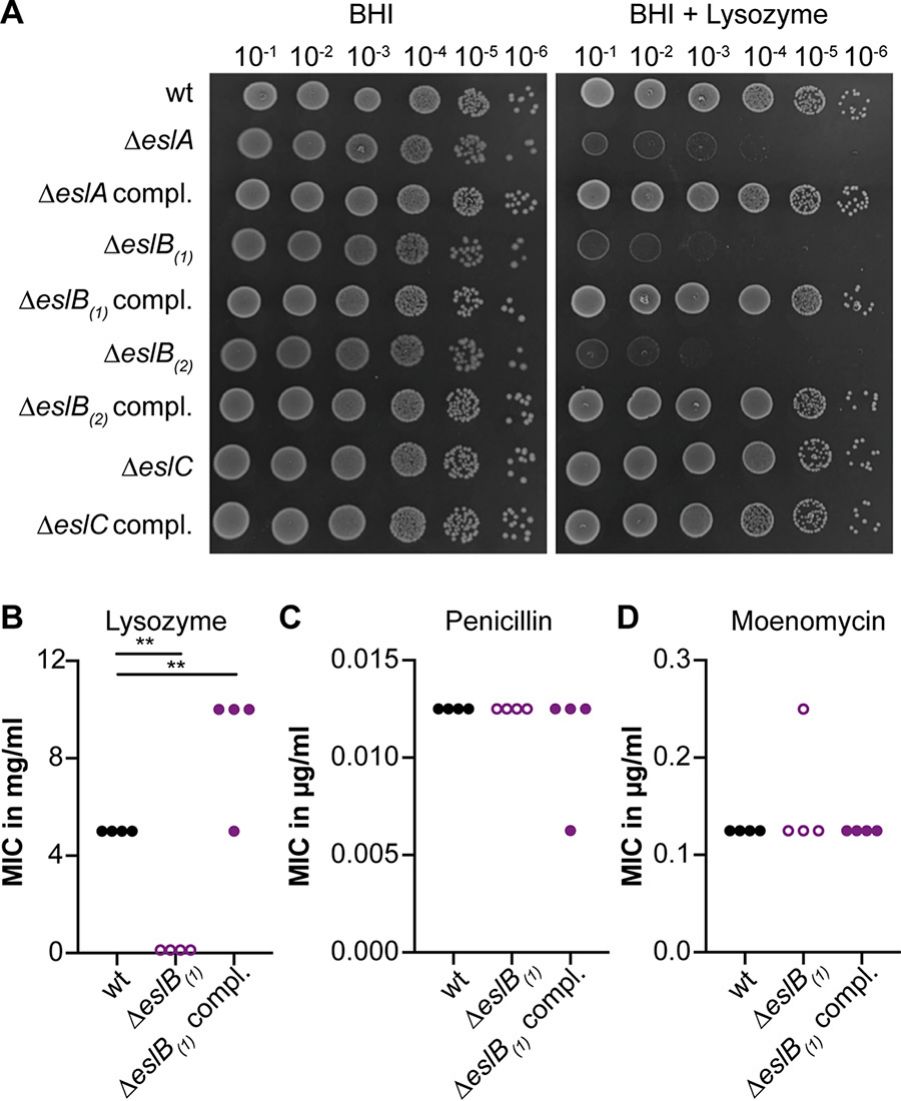
Impact of *eslB* deletion on resistance to cell wall-targeting antimicrobials. (A) Plate spotting assay. Dilutions of overnight cultures of L. monocytogenes strains 10403S (wild type [wt]), 10403SΔ*eslA*, 10403SΔ*eslA* compl., 10403SΔ*eslB*_1_, 10403SΔ*eslB*_1_ compl., 10403SΔ*eslB*_2_, 10403SΔ*eslB*_2_ compl., 10403SΔ*eslC*, and 10403SΔ*eslC* compl. were spotted on BHI plates and BHI plates containing 100 µg/ml lysozyme, both supplemented with 1 mM IPTG. A representative result from three independent experiments is shown. (B to D) MICs for L. monocytogenes strains 10403S (wt), 10403SΔ*eslB*_1_, and 10403SΔ*eslB*_1_ compl. of lysozyme (B), penicillin G (C), and moenomycin (D). Strain 10403SΔ*eslB*_1_ compl. was grown in the presence of 1 mM IPTG. The results of four independent experiments are shown. For statistical analysis, a one-way analysis of variance (ANOVA) followed by Dunnett’s multiple-comparison test was used (**, *P* ≤ 0.01).

An *eslA* in-frame deletion mutant and an *eslB* transposon insertion mutant were shown to be more sensitive to lysozyme than the wild-type strain (18, 19). However, it is still unknown how the function of an ABC transporter is linked to this phenotype. To investigate this further, strains with markerless in-frame deletions in *eslA*, *eslB*, and *eslC* were constructed in the L. monocytogenes 10403S strain background. First, the lysozyme resistance of these mutants was assessed using a plate spotting assay. The *eslA* and *eslB* mutants showed reduced growth on brain heart infusion (BHI) plates containing 100 µg/ml lysozyme compared to the wild type and the *eslA* and *eslB* complementation strains (Fig. 2A). On the other hand, no phenotype was observed for the *eslC* mutant (Fig. 2A). Since deletion of *eslA* and *eslB* resulted in a decreased lysozyme resistance, and an *eslA* mutant has already been characterized in previous work (19), we focused here on the characterization of the *eslB* deletion strain.

In the course of the study, we determined the genome sequence of the originally constructed *eslB* mutant (10403SΔ*eslB*_1_) by whole-genome sequencing (WGS) and identified an additional small deletion in *lmo2396*, coding for an internalin protein with a leucine-rich repeat (LRR) and a mucin-binding domain (see Table S3 in the supplemental material). While, to the best of our knowledge, the contribution of Lmo2396 to the growth and pathogenicity of L. monocytogenes has not yet been investigated, other internalins are important and well-established virulence factors (28, 29). Our WGS analysis also revealed a single point mutation in *lmo2342*, coding for a pseudouridylate synthase in the complementation strain 10403SΔ*eslB*_1_ compl. (Table S3). Since we identified an additional mutation in a gene coding for a potential virulence factor in the *eslB* mutant, we constructed a second independent *eslB* mutant, 10403SΔ*eslB*_2_. We also constructed a second complementation strain, strain 10403SΔ*eslB*_2_ *P_eslA_-eslABC* (or 10403SΔ*eslB*_2_ compl.), in which the *eslABC* genes are expressed from the native *eslA* promoter from a chromosomally integrated plasmid. Our WGS analysis revealed that strain 10403SΔ*eslB*_2_ did not contain any secondary mutations (Table S3). A 1-bp deletion in gene *lmo2022*, encoding a predicted NifS-like protein required for NAD biosynthesis, was identified in strain 10403SΔ*eslB*_2_ compl. (Table S3), which, if noncomplementable phenotypes are observed, needs to be kept in mind. We confirmed that our second *eslB* mutant strain, 10403SΔ*eslB*_2_, showed the same lysozyme sensitivity phenotype and that this phenotype could be complemented in strain 10403SΔ*eslB*_2_ compl., in which *eslB* is expressed along with *eslA* and *eslC* from its native promoter (Fig. 2A). Since we identified the genomic alterations only in the course of the study, some experiments were performed as stated with the original *eslB* mutant and complementation strains 10403SΔ*eslB*_1_ and 10403SΔ*eslB*_1_ compl., while other experiments were conducted with strains 10403SΔ*eslB*_2_ and 10403SΔ*eslB*_2_ compl.

Using broth microdilution assays, we observed a 40-fold-lower MIC for lysozyme for L. monocytogenes strain 10403SΔ*eslB*_1_ than for the wild-type strain (Fig. 2B; Fig. S1A) (18, 19). This phenotype could be complemented, and strain 10403SΔ*eslB*_1_ compl., in which *eslB* is expressed from an IPTG (isopropyl-β-d-thiogalactopyranoside)-inducible promoter, is slightly more resistant to lysozyme than the wild-type strain (Fig. 2B). Next, we tested whether the resistance to two cell wall-targeting antibiotics, namely, penicillin and moenomycin, is changed upon deletion of *eslB*. The MICs obtained for the wild-type, *eslB* deletion, and *eslB* complementation strains were comparable (Fig. 2C and D), suggesting that the deletion of *eslB* does not lead to a general sensitivity to all cell wall-acting antimicrobials but is specific to lysozyme.

**FIG 3:**
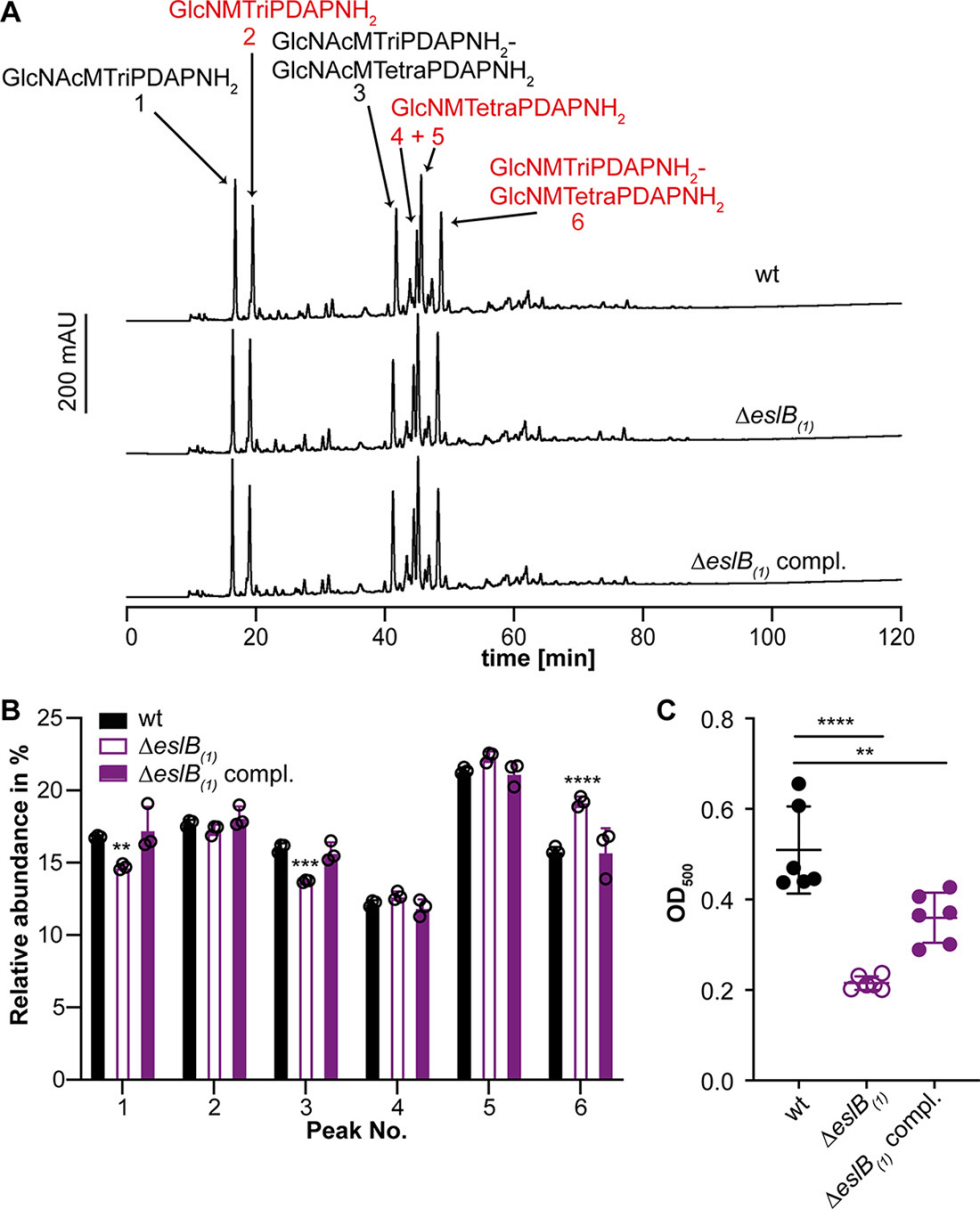
Deletion of *eslB* leads to changes in the peptidoglycan structure. (A) HPLC analysis of muropeptides derived from mutanolysin-digested peptidoglycan isolated from strains 10403S (wt), 10403SΔ*eslB*_1_, and 10403SΔ*eslB*_1_ compl. The muropeptide spectrum of the wild-type strain 10403S was published previously (43). Major muropeptide peaks are labeled and numbered 1 to 6 according to previously published HPLC spectra (18, 44), with labels in red corresponding to muropeptides with *N*-deacetylated GlcNAc residues and peaks 1 and 2 corresponding to monomeric and 4 to 6 to dimeric (cross-linked) muropeptide fragments. Muropeptide abbreviations: GlcNAc, *N*-acetylglucosamine; GlcN, glucosamine; M, *N*-acetylmuramic acid; TriPDAPNH_2_, l-alanyl-γ-d-glutamyl-amidated *meso*-diaminopimelic acid; TetraPDAPNH_2_, l-alanyl-γ-d-glutamyl-amidated *meso*-diaminopimelyl-d-alanine. (B) Quantification of the relative abundance of muropeptide peaks 1 to 6 for peptidoglycan isolated from strains 10403S (wt), 10403SΔ*eslB*_1_, and 10403SΔ*eslB*_1_ compl. For quantification, the sum of the peak areas was set to 100% and the area of individual peaks was determined. Average values and standard deviations were calculated from three independent peptidoglycan extractions and plotted. For statistical analysis, a two-way ANOVA followed by Dunnett’s multiple-comparison test was used (**, *P* ≤ 0.01; ***, *P* ≤ 0.001; ****, *P* ≤ 0.0001). (C) The degree of *O*-acetylation of purified peptidoglycan of strains 10403S (wt), 10403SΔ*eslB*_1_, and 10403SΔ*eslB*_1_ compl. was determined by a colorimetric assay as described in Materials and Methods. Average values and standard deviations were calculated from three independent peptidoglycan extractions and two technical repeats and plotted. For statistical analysis, a two-way ANOVA followed by Dunnett’s multiple-comparison test was used (**, *P* ≤ 0.01; ****, *P* ≤ 0.0001).

In L. monocytogenes, lysozyme resistance is achieved by the modification of the peptidoglycan (PG) by *N*-deacetylation via PgdA and *O*-acetylation via OatA (9, 10). To assess whether deletion of *eslB* affects the *N*-deacetylation and cross-linking of PG, PG was isolated from wild-type 10403S and the *eslB* deletion and complementation strains and digested with mutanolysin, and the muropeptides were analyzed by high-performance liquid chromatography (HPLC). This analysis revealed a slight increase in PG cross-linking in the *eslB* mutant strain (68% ± 0.53%) compared to the wild type (65.47% ± 0.31%) and the complementation strain grown in the presence of IPTG (64.57% ± 2.3%) (Fig. 3A and B). The GlcNAc residues of the PG isolated from the *eslB* deletion strain were also slightly more deacetylated (71.54% ± 0.21%) than those from the wild type (67.17% ± 0.31%) and the complementation strain (67% ± 2.27%) (Fig. 3A and B), which should theoretically result in an increase and not a decrease in lysozyme resistance.

While only *N*-deacetylation can be assessed by the PG analysis described above, *O*-acetylation has also been shown to contribute to lysozyme resistance. Indeed, in Neisseria gonorrhoeae and Proteus mirabilis, a correlation with the degree of *O*-acetylation and lysozyme resistance has been reported (30, 31). When we assessed the degree of *O*-acetylation using a colorimetric assay, the PG isolated from the *eslB* mutant was less *O*-acetylated than the wild type and the complementation strain, suggesting that reduced *O*-acetylation of the PG might contribute to the reduced lysozyme resistance of the *eslB* mutant strain (Fig. 3C). However, while the *eslB* complementation strain displayed slightly higher lysozyme resistance than the wild-type strain (Fig. 2B), the degree of *O*-acetylation was not fully restored to wild-type levels in the complementation strain. This suggests that additional factors besides *O*-acetylation might be altered in the *eslB* mutant.

### Deletion of *eslB* results in a growth defect in high-sugar media.

**FIG 4:**
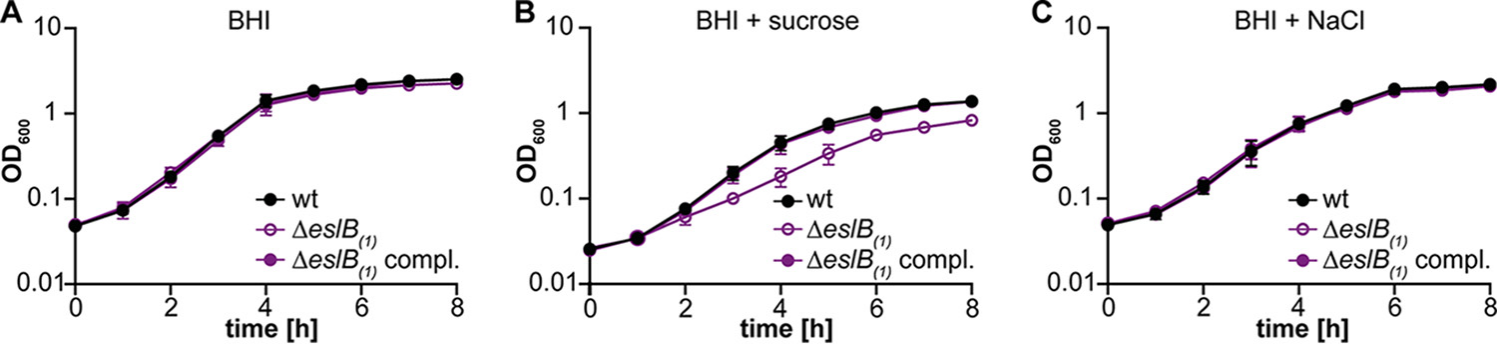
Addition of sucrose but not NaCl negatively impacts the growth of the L. monocytogenes *eslB* mutant strain. Bacterial growth curves are shown. L. monocytogenes strains 10403S (wt), 10403SΔ*eslB*_1_, and 10403SΔ*eslB*_1_ compl. were grown in BHI broth (A), BHI broth containing 0.5 M sucrose (B), or BHI broth containing 0.5 M NaCl (C). Strain 10403SΔ*eslB*_1_ compl. was grown in the presence of 1 mM IPTG. OD_600_ readings were determined at hourly intervals, and the average values and standard deviations from three independent experiments were calculated and plotted.

The bacterial cell wall is an important structure for maintaining cell integrity and preventing lysis due to high internal turgor pressure or changes in external osmolality. Alterations of the PG structure or other cell wall defects leading to impaired cell wall integrity could affect the growth of bacteria in environments with high osmolalities, e.g., in the presence of high salt or sugar concentrations. Next, we compared the growth of the wild type and the *eslB* mutant and complementation strains at 37°C in different media. No growth difference could be observed between the strains tested when they were grown in BHI medium (Fig. 4A; Fig. S1B). However, the *eslB* deletion strain grew more slowly in BHI medium containing 0.5 M sucrose than the wild type and the *eslB* complementation strain (Fig. 4B; Fig. S1C). A similar growth phenotype could be observed when the strains were grown in BHI medium containing either 0.5 M fructose, glucose, maltose, or galactose (Fig. S2). In contrast, the presence of 0.5 M NaCl did not affect the growth of the *eslB* deletion strain (Fig. 4C). These results suggest that the observed growth defect seen for the *eslB* mutant is not caused solely by the increase in external osmolality but rather seems to be specific to the presence of high concentrations of sugars.

### Deletion of *eslB* results in increased endogenous and lysozyme-induced lysis.

**FIG 5:**
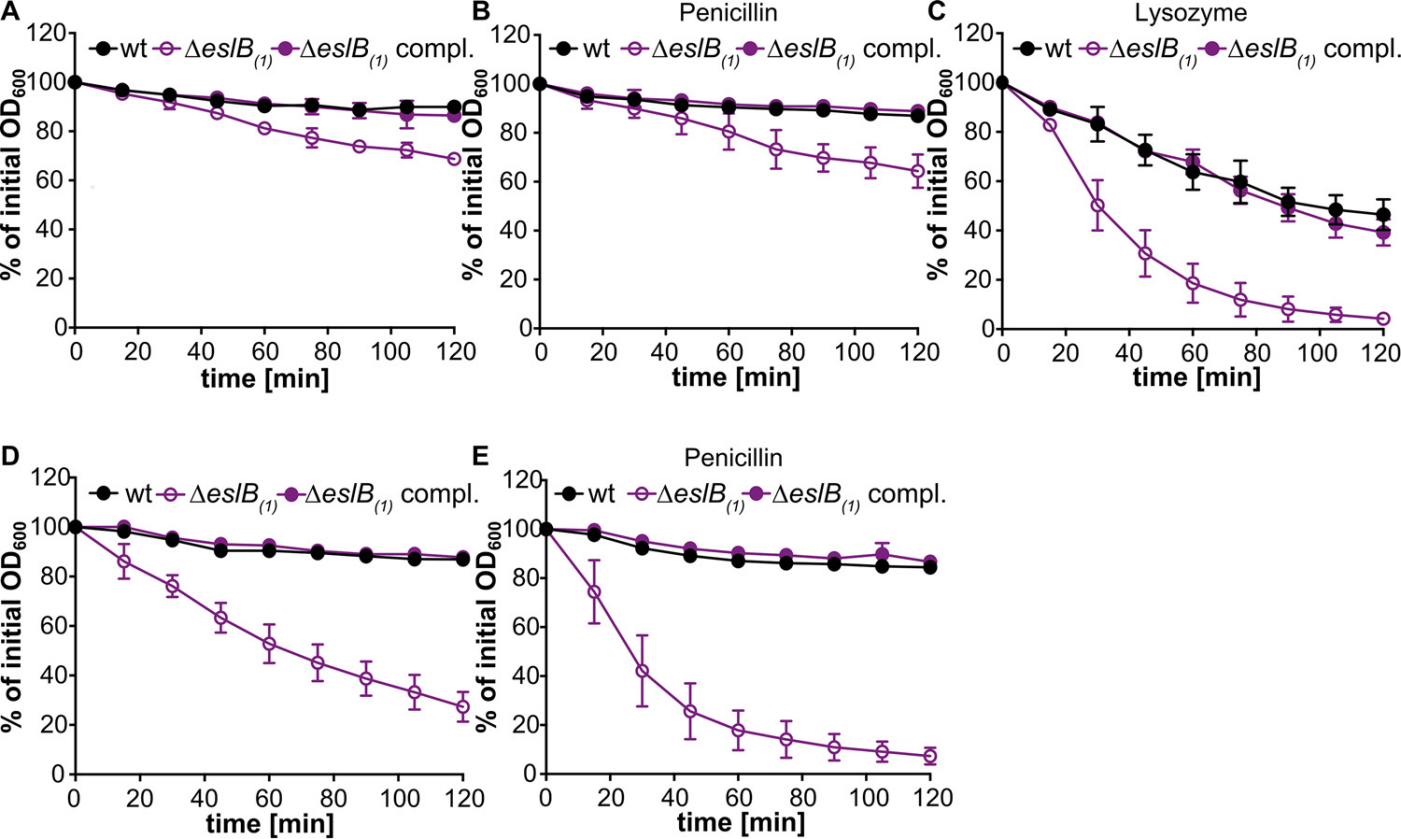
An L. monocytogenes *eslB* deletion strain shows increased endogenous and lysozyme-induced autolysis. Autolysis assays were performed with L. monocytogenes strains 10403S (wt), 10403SΔ*eslB*_1_, and 10403SΔ*eslB*_1_ compl. Bacteria were grown for 4 h in BHI medium (A to C) or in BHI medium containing 0.5 M sucrose (containing 1 mM IPTG for 10403SΔ*eslB*_1_ compl.) (D to E), and subsequently, bacterial suspensions were prepared in 50 mM Tris HCl, pH 8 (A and D), 50 mM Tris HCl, pH 8, containing 25 µg/ml penicillin (B and E), or 2.5 µg/ml lysozyme (C). Cell lysis was followed by taking OD_600_ readings every 15 min. The initial OD_600_ reading for each bacterial suspension was set to 100% and subsequent readings are shown as a percentage of the initial OD_600_ reading. The average OD_600_ percentages and standard deviations were calculated from three independent experiments and plotted.

The observed lysozyme sensitivity and the growth defect of the *eslB* deletion strain in media containing high concentrations of sucrose raised the question of whether the absence of EslB might also cause an impaired cell wall integrity and an increased autolysis due to this impairment. To test this, autolysis assays were performed. To this end, the L. monocytogenes wild-type strain 10403S and the *eslB* deletion and complementation strains were grown in BHI medium and subsequently transferred to Tris-HCl buffer (pH 8). After 2 h of incubation at 37°C, the optical density at 600 nm (OD_600_) of the suspensions of the wild type and *eslB* complementation strain had dropped to 89.9% ± 1.6% and 86.5% ± 2.9% of the initial OD_600_, respectively (Fig. 5A). Enhanced endogenous cell lysis was observed for the *eslB* mutant strain and the OD_600_ of the suspensions dropped to 68.8% ± 1.7% of the initial OD_600_ within 2 h (Fig. 5A). The addition of penicillin had no impact on the cell lysis of any of the strains tested (Fig. 5B). On the other hand, the addition of 2.5 µg/ml lysozyme increased the rate of cell lysis of all strains but had a particularly drastic effect on the *eslB* mutant. After 30 min, the OD_600_ reading of a suspension of the *eslB* deletion strain had dropped to 50.3% ± 10.2% of the initial OD_600_. For the wild-type and *eslB* complementation strains, it took 90 min to see a 50% reduction in the OD_600_ readings (Fig. 5C).

**FIG 6:**
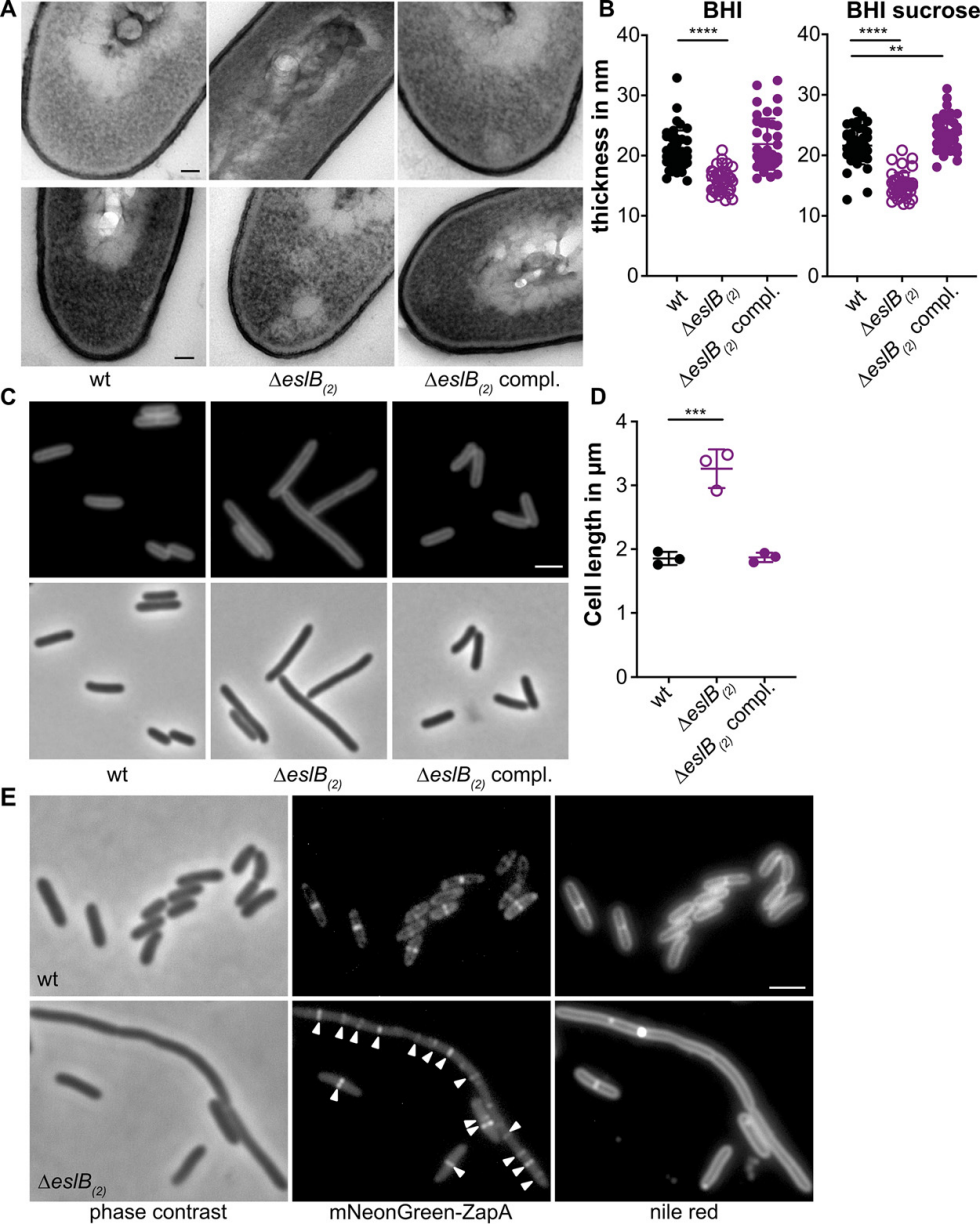
L. monocytogenes *eslB* mutant bacteria produce a thinner cell wall, have a cell division defect, and have an increased cell length. (A) Transmission electron microscopy images. Ultrathin-sectioned cells of L. monocytogenes strains 10403S (wt), 10403SΔ*eslB*_2_, and 10403SΔ*eslB*_2_ compl. were subjected to transmission electron microscopy after growth in BHI broth (top) or BHI broth containing 0.5 M sucrose (bottom). Bar, 50 nm. Representative images from two independent experiments are shown. (B) Cell wall thickness. Per growth condition, cell wall thickness of 40 cells was measured at three different locations, and average values were plotted. For statistical analysis, a two-way ANOVA followed by Dunnett’s multiple-comparison test was used (**, *P* ≤ 0.01; ****, *P* ≤ 0.0001). (C) Microscopy images of L. monocytogenes strains 10403S (wt), 10403SΔ*eslB*_2_, and 10403SΔ*eslB*_2_ compl. Bacterial membranes were stained with Nile red, and cells were analyzed by phase-contrast and fluorescence microscopy. Bar, 2 µm. Representative images from three independent experiments are shown. (D) Cell length of L. monocytogenes strains 10403S (wt), 10403SΔ*eslB*_2_, and 10403SΔ*eslB*_2_ compl. The lengths of 300 cells per strain were measured, and the median cell length was calculated. Three independent experiments were performed, and the average values and standard deviations of the median cell length were plotted. For statistical analysis, a one-way ANOVA followed by Dunnett’s multiple-comparison test was used (***, *P* ≤ 0.001). (E) Localization of mNeonGreen-ZapA in L. monocytogenes strains 10403S (wt) and 10403SΔ*eslB*_2_. Bacterial membranes were stained with Nile red, and cells were analyzed by phase-contrast microscopy (left) and by fluorescence microscopy to detect mNeonGreen (middle) and Nile red fluorescence (right) signals. Arrowheads highlight ZapA foci in cells of the L. monocytogenes *eslB* mutant. Bar, 2 µm. Representative images from three independent experiments are shown.

Next, we wanted to determine what impact the growth in the presence of high levels of sucrose has on endogenous bacterial autolysis rates. To this end, the wild-type 10403S and the *eslB* mutant and complementation strains were grown in BHI medium supplemented with 0.5 M sucrose, and cell suspensions were prepared in Tris-buffer and used in autolysis assays. While the wild-type and complementation strain showed autolysis rates following growth in BHI-sucrose medium (Fig. 5D) similar to those seen after growth in BHI medium (Fig. 5A), the *eslB* mutant lysed rapidly following growth in BHI–0.5 M sucrose medium (Fig. 5E). The lysis of the *eslB* mutant strain could be further enhanced by the addition of 25 µg/ml penicillin, a concentration which is only bacteriostatic for the wild-type L. monocytogenes strain 10403S (Fig. 5E). These findings indicate that the *eslB* mutant is sensitive to osmotic downshifts, and we thus wondered whether, in addition to the changes in the PG modifications and cross-linking, more general differences in the ultrastructure of the cell wall might be observed. To test this, cells of L. monocytogenes strains 10403S, 10403SΔ*eslB*_2_, and 10403SΔ*eslB*_2_ compl. were subjected to transmission electron microscopy. The *eslB* deletion strain produced a thinner PG layer, 15.8 ± 1.9 nm, when grown in BHI broth than the wild type (20 ± 3.4 nm) and the complementation strain (20 ± 4.3 nm) (Fig. 6A and B). This phenotype was even more pronounced when the strains were grown in BHI broth containing 0.5 M sucrose. The PG layer of the *eslB* mutant had a thickness of 15 ± 2 nm, while the wild type and the complementation strain produced PG layers of 21.4 ± 3.1 and 23.3 ± 2.8 nm, respectively (Fig. 6A and B). We hypothesize that the enhanced endogenous lysis as well as the lysozyme sensitivity of the *eslB* mutant is likely caused by a thinner PG layer combined with the observed alterations in PG structure, such as reduced *O*-acetylation.

### The *eslB* deletion strain is impaired in cell division but not in virulence.

The increased endogenous autolysis together with the observed changes in the PG structure of the *eslB* deletion strain could result in an increased sensitivity to autolysins. The major autolysins of L. monocytogenes are p60 and NamA, which hydrolyze PG and are required for daughter cell separation during cell division (32, 33). Absence of either p60 or NamA results in the formation of chains (32, 33). We thus wondered whether deletion of *eslB* causes changes in the cell morphology of L. monocytogenes. Microscopic analysis revealed that cells lacking EslB are significantly longer, with a median cell length of 3.26 ± 0.25 µm, than the L. monocytogenes wild-type strain, which produced cells with a length of 1.85 ± 0.08 µm (Fig. 6C and D), highlighting that the absence of EslB results in a cell division defect. To test whether the assembly of the early divisome is affected by the absence of EslB, we compared the localization of the early cell division protein ZapA in the wild type and the *eslB* mutant background. In L. monocytogenes wild-type cells, a signal was observed at midcell for cells which had initiated the division process (Fig. 6E). While short cells of the *eslB* mutant also possess only a single fluorescent signal, several ZapA fluorescence foci could be observed in elongated cells (Fig. 6E), suggesting that early cell division proteins can still localize in the *eslB* mutant and that a process downstream seems to be perturbed in the absence of EslB.

**FIG 7:**
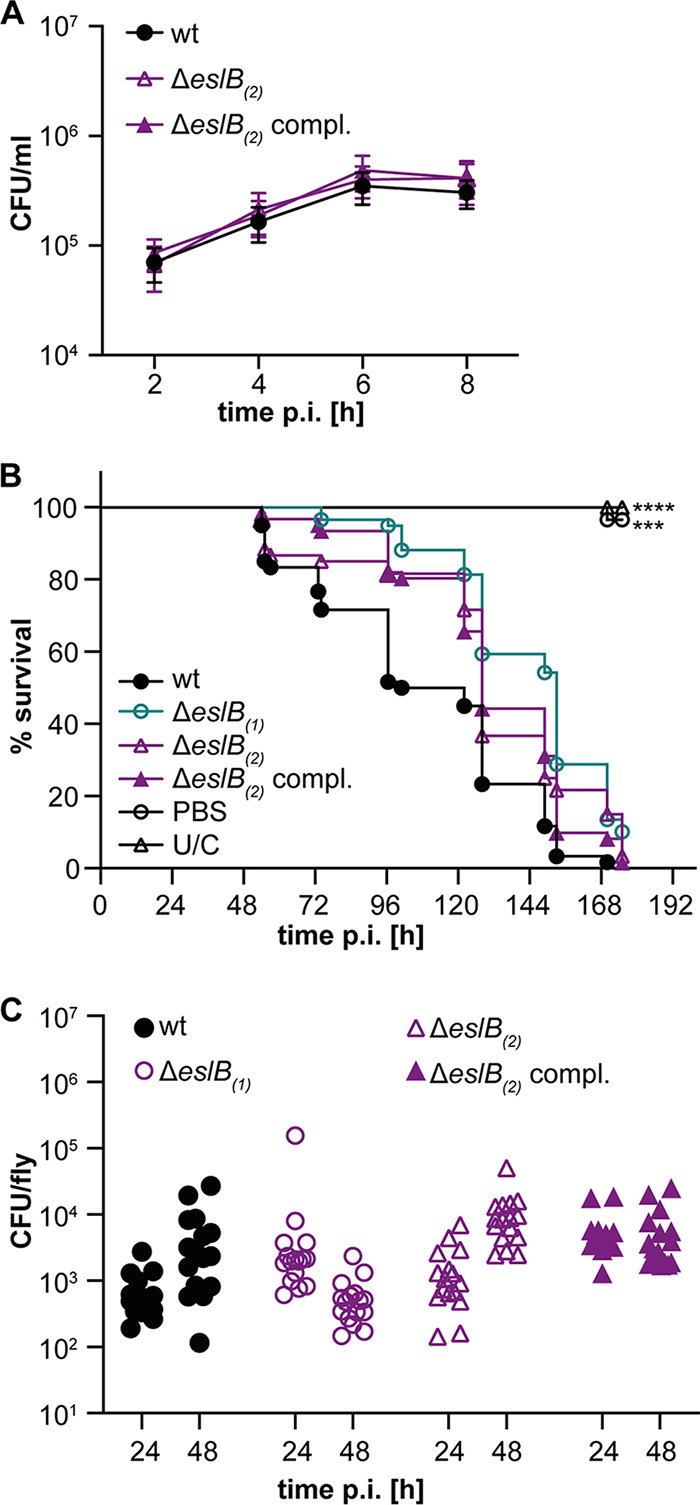
Impact of deletion of *eslB* on the virulence of L. monocytogenes. (A) Intracellular growth of L. monocytogenes strains 10403S (wt), 10403SΔ*eslB*_2_, and 10403SΔ*eslB*_2_ compl. in mouse bone marrow-derived macrophages (BMMs). The infection assay was performed as described in Materials and Methods. The average number of CFU per milliliter and standard deviations from three independent experiments were calculated and plotted. (B) Survival curve of flies infected with L. monocytogenes. Flies were infected with L. monocytogenes strains 10403S (wt), 10403SΔ*eslB*_1_, 10403SΔ*eslB*_2_, and 10403SΔ*eslB*_2_ compl. Uninjected control flies (U/C) and flies injected with PBS were used as controls. Fly death was monitored daily. For statistical analysis, a one-way ANOVA followed by Dunnett’s multiple-comparison test was used (***, *P* ≤ 0.001; ****, *P* ≤ 0.0001). (C) Bacterial quantification. Sixteen flies infected with the indicated L. monocytogenes strain were collected 24 and 48 h postinfection, and bacterial load (CFU) was determined as described in Materials and Methods. For statistical analysis, a nested one-way ANOVA followed by Dunnett’s multiple-comparison test was used. The observed differences were not statistically significant.

Next, we wanted to assess whether the impaired cell integrity and the observed cell division defect would also affect the virulence of the L. monocytogenes *eslB* mutant. Of note, in a previous study, it was shown that deletion of *eslA*, coding for the ATP-binding protein component of the ABC transporter, has no effect on the cell-to-cell spread of L. monocytogenes (19). To determine whether EslB is involved in the virulence of L. monocytogenes, primary mouse macrophages were infected with wild-type 10403S, the *eslB* mutant 10403SΔ*eslB*_2_, and the complementation strain 10403SΔ*eslB*_2_ compl. All three strains showed a comparable intracellular growth pattern (Fig. 7A), suggesting that EslB does not impact the ability of L. monocytogenes to grow in primary mouse macrophages. Next, we assessed the ability of the *eslB* deletion strains to kill Drosophila melanogaster, as lysozyme is one important component of its innate immune response (34). All uninfected flies and 96.6% of the flies that were injected with phosphate-buffered saline (PBS) survived for the duration of the experiment (Fig. 7B). No statistically significant differences in the survival and bacterial load of flies infected with the different L. monocytogenes strains were observed (Fig. 7B and C). These results indicate that, while EslB does not impact the ability of L. monocytogenes to infect and kill mammalian macrophages or Drosophila melanogaster, it nonetheless impacts the cell division and cell wall integrity of L. monocytogenes, and consistent with this, we have identified changes in the composition and thickness of the peptidoglycan layer.

## DISCUSSION

Over the past years, several determinants contributing to the intrinsic lysozyme resistance of L. monocytogenes have been described (9, 10, 18, 19). One of these is a predicted ABC transporter encoded by the *eslABCR* operon (18, 19). In this study, we aimed to further investigate the role of the ABC transporter EslABC in lysozyme resistance of L. monocytogenes. Using bacterial two-hybrid assays, we could show that EslB and EslC interact with each other, and hence, it is tempting to speculate that the transmembrane component of the ABC transporter consists of a heterodimer of EslB and EslC. However, analysis of different deletion mutants revealed that only EslA and EslB are required for lysozyme resistance of L. monocytogenes, suggesting that EslC is not required for the function of the ABC transporter under our assay conditions. Surprisingly, we did not observe an interaction between EslA and EslB using bacterial two-hybrid assays; thus, further experiments are required to determine the composition of the ABC transporter and its interaction partners.

Next, we analyzed the PG structure of the *eslB* deletion strain and found that the PG isolated from the *eslB* mutant was slightly more cross-linked and also that the fraction of deacetylated GlcNAc residues was slightly increased compared to the PG isolated from the wild-type strain 10403S. Deacetylation of GlcNAc residues in PG is achieved by the *N*-deacetylase PgdA and has been shown to lead to increased lysozyme resistance (9). Since we saw a slight increase in the deacetylation of GlcNAc residues in the *eslB* mutant strain, our results indicate that the lysozyme sensitivity phenotype of the *eslB* deletion strain is independent of PgdA and that this enzyme functions properly in the mutant strain. A second enzyme required for lysozyme resistance in L. monocytogenes is OatA, which transfers *O*-acetyl groups to MurNAc (10, 35, 36). Using a colorimetric *O*-acetylation assay, we were able to show that PG isolated from the *eslB* mutant is less *O*-acetylated. In addition, transmission electron microscopy revealed that the *eslB* mutant produces a thinner PG layer, and we assume that this and the reduction in *O*-acetylation contribute to the lysozyme sensitivity of strain 10403SΔ*eslB*.

Growth comparisons in different media revealed that the absence of EslB results in reduced growth in BHI broth containing high concentrations of mono- or disaccharides. One could speculate that the EslABC transporter might be a sugar transporter with a broad sugar spectrum. However, we could not identify a potential substrate-binding protein encoded in the *esl* operon, which is important for substrate recognition and delivery to ABC importers. EslABC could also be involved in the export of PG components and thus affect cell wall biosynthesis in L. monocytogenes. Indeed, we could show that the *eslB* mutant produces a thinner PG layer than the wild-type strain, suggesting that EslABC affects PG biosynthesis. Future studies will aim to determine how the ABC transporter EslABC influences the biosynthesis and subsequent modification of PG in L. monocytogenes.

The absence of EslB leads to the formation of elongated cells; however, it is currently not clear how the function of EslABC is linked to cell division of L. monocytogenes. It seems unlikely that the activity or levels of the autolysins p60 and NamA are affected by the absence of EslB. While *iap* and *namA* mutants also form chains of cells, the cell length of individual cells is still similar to that of wild-type cells; however, the bacteria are just unable to separate (32, 33, 37). This is in contrast to the *eslB* mutant, in which the cell length of individual cells is increased, suggesting that cell division is blocked at an earlier step. In elongated cells of the *eslB* mutant, we could observe several ZapA foci, suggesting that really early cell division proteins can still be recruited in this strain. Thus, a process downstream of ZapA localization but before the construction of the actual cell septum is perturbed in the absence of EslB. EslABC could potentially affect the activity of cell division proteins or the localization of late cell division-specific proteins. Hence, deletion of *eslB* could lead to a delayed assembly of an active divisome, which could lead to an altered PG biosynthesis at the division site and an impaired cell integrity. Indeed, cells of the *eslB* mutant lysed more rapidly than the L. monocytogenes wild-type strain 10403S when shifted from BHI broth to Tris buffer. The autolysis of cells lacking EslB was strongly induced following growth in BHI supplemented with 0.5 M sucrose prior to the incubation in Tris buffer. These results indicate that the *eslB* mutant is sensitive to an osmotic downshift, and we hypothesize that this is due to the production of a thinner PG layer and a resulting impairment of cell integrity.

Reduced lysozyme resistance is often associated with reduced virulence. An E. faecalis strain with a deletion in the gene coding for the peptidoglycan deacetylase PgdA showed a reduced ability to kill Galleria mellonella (11). Similarly, an S. pneumoniae *pgdA* mutant showed decreased virulence in a mouse model of infection (13). In our study, we found that inactivation of EslB does not affect the intracellular growth of L. monocytogenes in primary mouse macrophages or the ability to kill Drosophila melanogaster. These observations are consistent with a previous report that another component of the EslABC transporter, EslA, is dispensable for the ability of L. monocytogenes to spread from cell to cell (19). Previously, it was also shown that combined inactivation of PgdA and OatA reduced the ability of L. monocytogenes to grow in bone-marrow derived macrophages, whereas inactivation of PgdA alone had no impact on the virulence of L. monocytogenes (35). We therefore reason that the changes in PG structure and associated reduction in lysozyme resistance caused by deletion of *eslB* are not sufficient to affect the ability of L. monocytogenes to grow and survive in primary macrophages and flies.

Taken together, our results show that not only is EslB important for resistance of L. monocytogenes to lysozyme but also its absence affects the organism’s autolysis, cell division, and ability to grow in media containing high concentrations of sugars. Our results indicate that the ABC transporter EslABC has a direct or indirect impact on peptidoglycan biosynthesis and maintenance of cell integrity in L. monocytogenes.

## MATERIALS AND METHODS

### Bacterial strains and growth conditions.

All strains and plasmids used in this study are listed in Table S1 in the supplemental material. Escherichia coli strains were grown in Luria-Bertani (LB) medium and Listeria monocytogenes strains were grown in brain heart infusion (BHI) medium at 37°C unless otherwise stated. If necessary, antibiotics and supplements were added to the medium at the following concentrations: for E. coli cultures, ampicillin (Amp) at 100 µg/ml, chloramphenicol (Cam) at 20 µg/ml, and kanamycin (Kan) at 30 µg/ml; for L. monocytogenes cultures, Cam at 10 µg/ml, erythromycin (Erm) at 5 µg/ml, Kan at 30 µg/ml, nalidixic acid (Nal) at 20 µg/ml, streptomycin (Strep) at 200 µg/ml, and IPTG at 1 mM.

### Strain and plasmid construction.

All primers used in this study are listed in Table S2. For the markerless in-frame deletion of *lmo2768* (*lmrg_01927*; *eslB*), approximately 1-kb DNA fragments up- and downstream of the *eslB* gene were amplified by PCR using the primer pairs ANG2532/ANG2533 and ANG2534/ANG2535. The resulting PCR products were fused in a second PCR using the primer pair ANG2532/ANG2535, and the product was cut with BamHI and XbaI and ligated with plasmid pKSV7 that had been cut with the same enzymes. The resulting plasmid, pKSV7-Δ*eslB*, was recovered in E. coli XL1-Blue, yielding strain ANG4236. The plasmid was subsequently transformed into L. monocytogenes strain 10403S, and *eslB* was deleted by allelic exchange using a previously described procedure (38). The deletion of *eslB* was verified by PCR. The deletion procedure was performed with two independent transformants and resulted in the construction of two independent *eslB* mutant strains, 10403SΔ*eslB*_1_ (ANG4275) and 10403SΔ*eslB*_2_ (ANG5662).

For complementation analysis, pIMK3-*eslB* was constructed, in which the expression of *eslB* can be induced by IPTG. The *eslB* gene was amplified using the primer pair ANG2812/ANG2813, and the product was cut with NcoI and SalI and fused with pIMK3 that had been cut with the same enzymes. The resulting plasmid pIMK3-*eslB* was recovered in E. coli XL1-Blue yielding strain ANG4647. Due to difficulties in preparing electrocompetent cells of L. monocytogenes *eslB* mutant strains, plasmid pIMK3-*eslB* was first electroporated into the wild-type L. monocytogenes strain 10403S, yielding strain 10403S pIMK3-*eslB* (ANG4678). In the second step, *eslB* was deleted from the genome of strain ANG4678 resulting in the construction of the first *eslB* complementation strain 10403SΔ*eslB*_1_ pIMK3-*eslB* (ANG4688; 10403SΔ*eslB*_1_ compl.). In addition, complementation plasmid pPL3e-P*_eslA_*-*eslABC* was constructed. To this end, the *eslABC* genes, including the upstream promoter region, were amplified by PCR using primer pair ANG3349/ANG3350. The resulting PCR product was cut with SalI and BamHI and fused with plasmid pPL3e that had been cut with the same enzymes. Plasmid pPL3e-P*_eslA_*-*eslABC* was recovered in E. coli XL1-Blue, yielding strain ANG5660. Next, plasmid pPL3e-P*_eslA_*-*eslABC* was transformed into E. coli SM10, yielding strain ANG5661. Last, SM10 pPL3e-P*_eslA_*-*eslABC* was used as a donor strain to transfer plasmid pPL3e-P*_eslA_*-*eslABC* by conjugation into L. monocytogenes strain 10403SΔ*eslB*_2_ (ANG5662) using a previously described method (39). This resulted in the construction of the second *eslB* complementation strain, 10403SΔ*eslB*_2_ pPL3e-P*_eslA_*-*eslABC* (ANG5663; 10403SΔ*eslB*_2_ compl.).

For the markerless in-frame deletion of *lmo2769* (*lmrg_01926*; *eslA*), and *lmo2767* (*lmrg_01928*; *eslC*), approximately 1-kb DNA fragments up- and downstream of the corresponding gene were amplified by PCR using primer pairs LMS160/LMS161 and LMS159/LMS162 (*eslA*) and LMS155/LMS158 and LMS156/LMS157 (*eslC*). The resulting PCR products were fused in a second PCR using primers LMS159/LMS160 (*eslA*) and LMS155/LMS156 (*eslC*). The products were cut with BamHI and EcoRI (*eslA*) and BamHI and KpnI (*eslC*) and ligated with plasmid pKSV7 that had been cut with the same enzymes. The resulting plasmids, pKSV7-Δ*eslA* and pKSV7-Δ*eslC*, were recovered in E. coli XL1-Blue, yielding strains EJR54 and EJR43, respectively. The plasmids were subsequently transformed into L. monocytogenes strain 10403S, and *eslA* and *eslC* were deleted by allelic exchange, yielding strains 10403SΔ*eslA* (LJR33) and 10403SΔ*eslC* (LJR7). Plasmid pPL3e-P*_eslA_*-*eslABC* was transferred into LJR33 and LJR7 via conjugation using strain SM10 pPL3e-P*_eslA_*-*eslABC* (ANG5661) as a donor strain, yielding strains 10403SΔ*eslA* pPL3e-P*_eslA_*-*eslABC* (LJR34, short 10403SΔ*eslA* compl.) and 10403SΔ*eslC* pPL3e-P*_eslA_*-*eslABC* (LJR21; 10403SΔ*eslC* compl.).

For the construction of bacterial two hybrid plasmids, *eslA*, *eslB*, and *eslC* were amplified by PCR using primer pairs JR44/JR45, JR46/JR47, and JR48/JR49, respectively. The resulting *eslA* and *eslC* fragments were cut with XbaI and KpnI and ligated into pKT25, pKNT25, pUT18, and pUT18C that had been cut with the same enzymes. The *eslB* fragment was cut with XbaI and BamHI and ligated into XbaI/BamHI-cut pKT25, pKNT25, pUT18, and pUT18C. The resulting plasmids were recovered in E. coli XL1-Blue, yielding strains XL1-Blue pKNT25-*eslA* (EJR4), XL1-Blue pKT25-*eslA* (EJR5), XL1-Blue pUT18-*eslA* (EJR6), XL1-Blue pUT18C-*eslA* (EJR7), XL1-Blue pKNT25-*eslB* (EJR8), XL1-Blue pKT25-*eslB* (EJR9), XL1-Blue pUT18-*eslB* (EJR10), XL1-Blue pUT18C-*eslB* (EJR11), XL1-Blue pKNT25-*eslC* (EJR12), XL1-Blue pKT25-*eslC* (EJR13), and XL1-Blue pUT18C-*eslC* (EJR15). Using this approach, we were unable to construct pUT18-*eslC* without acquiring mutations in *eslC*. In a second attempt to generate pUT18-*eslC*, plasmid pKT25-*eslC* (from strain EJR13) was cut with XbaI and KpnI, and the *eslC* fragment was extracted and ligated into XbaI/KpnI-cut pUT18. The resulting plasmid was recovered in E. coli CLG190, yielding strain CLG190 pUT18-*eslC* (EJR14).

For the localization of an early cell division protein, mNeonGreen was fused to the N terminus of ZapA. For this purpose, the mNeonGreen gene and *zapA* were amplified using primer pairs JR73/JR39 and JR40/JR74, respectively. The resulting PCR products were fused in a second PCR using primers JR73/JR74, and the product was cut with NcoI and SalI and ligated with pIMK2 that had been cut with the same enzymes. pIMK2-*mNeonGreen*-*zapA* was recovered in E. coli XL1-Blue and transformed into E. coli S17-1, yielding strains EJR39 and EJR60, respectively. S17-1 pIMK2-*mNeonGreen*-*zapA* was used as a donor strain to transfer the plasmid pIMK2-*mNeonGreen*-*zapA* by conjugation into L. monocytogenes strains 10403S (ANG1263) and 10403SΔ*eslB*_2_ (ANG5662), resulting in the construction of strains 10403S pIMK2-*mNeonGreen*-*zapA* (LJR28) and 10403SΔ*eslB*_2_ pIMK2-*mNeonGreen*-*zapA* (LJR29).

### Bacterial two-hybrid assays.

Interactions between EslA, EslB and EslC were analyzed using bacterial adenylate cyclase two-hybrid (BACTH) assays (40). For this purpose, 15 ng of the indicated pKT25/pKNT25 and pUT18/pUT18C derivatives were cotransformed into E. coli strain BTH101. Transformants were spotted on LB agar plates containing 25 µg/ml kanamycin, 100 µg/ml ampicillin, 0.5 mM IPTG, and 80 µg/ml X-Gal (5-bromo-4-chloro-3-indolyl-β-d-galactopyranoside), and the plates were incubated at 30°C. Images were taken after an incubation of 48 h.

### Whole-genome sequencing.

Genomic DNA of L. monocytogenes was extracted using a FastDNA kit (MP Biomedicals), and libraries for sequencing were prepared using an Illumina Nextera DNA kit. The samples were sequenced at the London Institute of Medical Sciences using an Illumina MiSeq instrument and a 150 paired-end Illumina kit. The reads were trimmed and mapped to the L. monocytogenes 10403S reference genome (NC_017544), and single nucleotide polymorphisms (SNPs) with a frequency of at least 80% and small deletions (zero coverage) were identified using the CLC Genomics Workbench (Qiagen).

### Growth analysis.

L. monocytogenes strains were grown overnight in 5 ml BHI medium at 37°C with shaking. The next day, these cultures were used to inoculate 15 ml fresh BHI medium or BHI medium containing 0.5 M sucrose, fructose, glucose, maltose, galactose, or sodium chloride to an OD_600_ of 0.05. The cultures were incubated at 37°C with shaking at 180 rpm, OD_600_ readings were taken every hour for 8 h.

### Determination of MIC.

The MICs of the cell wall-acting antibiotics penicillin and moenomycin and the cell wall hydrolase lysozyme were determined in 96-well plates using a broth microdilution assay. Approximately 10^4^ L. monocytogenes cells were used to inoculate 200 µl BHI containing 2-fold dilutions of the different antimicrobials. The starting antibiotic concentrations were 0.025 µg/ml for penicillin G, 0.2 µg/ml for moenomycin and 10 mg/ml or 0.25 mg/ml for lysozyme. The 96-well plates were incubated at 37°C with shaking at 500 rpm in a plate incubator (Thermostar; BMG Labtech), and OD_600_ readings were determined after 24 h of incubation. The MIC was defined as the antibiotic concentration at which bacterial growth was inhibited by >90%.

### Plate spotting assay.

Overnight cultures of the indicated L. monocytogenes strains were adjusted to an OD_600_ of 1 and serially diluted to 10^−6^. A 5-µl portion of each dilution was spotted on BHI agar plates or BHI agar plates containing 100 µg/ml lysozyme, both containing 1 mM IPTG. Images of the plates were taken after incubation for 20 to 24 h at 37°C.

### Peptidoglycan isolation and analysis.

Overnight cultures of 10403SΔ*eslB*_1_ and 10403SΔ*eslB*_1_ compl. were diluted in 1 liter of BHI broth (supplemented with 1 mM IPTG for strain 10403SΔ*eslB*_1_ compl.) to an OD_600_ of 0.06 and incubated at 37°C. At an OD_600_ of 1, bacterial cultures were cooled on ice for 1 h, and the bacteria were subsequently collected by centrifugation. The peptidoglycan was purified and digested with mutanolysin, and the muropeptides were analyzed by HPLC using an Agilent 1260 infinity system, as previously described (41, 42). Peptidoglycan of the wild-type L. monocytogenes strain 10403S was purified and analyzed in parallel. The chromatogram of the same wild-type control strain was recently published (43) and was also used as part of this study, since all strains were analyzed at the same time. Major peaks 1 to 6 were assigned according to previously published HPLC spectra (18, 44), with peaks 2, 4, 5, and 6 corresponding to *N*-deacetylated GlcNAc residues. Peaks 1 and 2 correspond to monomeric and peaks 4 to 6 to dimeric (cross-linked) muropeptide fragments. Agilent Technology ChemStation software was used to integrate the areas of the main muropeptide. For quantification, the sum of the peak areas was set to 100% and the area of individual peaks was determined. The sum of values for peaks 3 to 6 corresponds to the percentage of cross-linking, whereas the deacetylation state was calculated by adding the values for peaks 4, 5, and 6. Averages values and standard deviations were calculated from three independent extractions.

### *O*-Acetylation assay.

Peptidoglycan of strains 10403S, 10403SΔ*eslB*_1_, and 10403SΔ*eslB*_1_ compl., which had not been treated with hydrofluoric acid and alkaline phosphatase to avoid removal of the *O*-acetyl groups, was used for the *O*-acetylation assays. *O*-Acetylation was measured colorimetrically according to the Hestrin method described previously (45) with slight modifications. Briefly, 800 µg of PG (dissolved in 500 µl H_2_O) was incubated with an equal volume of 0.035 M hydroxylamine chloride in 0.75 M NaOH for 10 min at 25°C. Next, 500 µl of 0.6 M perchloric acid and 500 µl of 70 mM ferric perchlorate in 0.5 M perchloric acid were added. The color change resulting from the presence of *O*-acetyl groups was quantified at 500 nm. An assay reaction with 500 µl H_2_O was used as a blank for the absorbance measurement.

### Autolysis assays.

L. monocytogenes strains were diluted in BHI medium alone or BHI medium supplemented with 0.5 M sucrose to an OD_600_ of 0.05 and grown for 4 h at 37°C. Cells were collected by centrifugation and resuspended in 50 mM Tris-HCl, pH 8, to an OD_600_ of 0.7 to 0.9 and incubated at 37°C. For penicillin- and lysozyme-induced lysis, 25 µg/ml penicillin G or 2.5 µg/ml lysozyme was added to the cultures. Autolysis was followed by determining OD_600_ readings every 15 min.

### Fluorescence and phase-contrast microscopy.

Overnight cultures of the indicated L. monocytogenes strains were diluted 1:100 in BHI medium and grown for 3 h at 37°C. For staining of the bacterial membrane, 100 µl of these cultures was mixed with 5 µl of 100 µg/ml Nile red solution and incubated for 20 min at 37°C. The cells were washed twice with PBS and subsequently suspended in 50 µl of PBS. Portions (1 to 1.5 µl) of the different samples were subsequently spotted on microscope slides covered with a thin agarose film (1.5% agarose in distilled water), air dried, and covered with a coverslip. Phase-contrast and fluorescence images were taken at a magnification of ×1,000 using a Zeiss Axio Imager.A1 microscope coupled to an AxioCam MRm camera and processed using the Zen 2012 software (blue edition). The Nile red fluorescence signal was detected using the Zeiss filter set 00. The lengths of 300 cells were measured for each experiment, and the median cell length was calculated.

For ZapA localization studies, overnight cultures of the indicated L. monocytogenes strains were grown in BHI medium at 37°C to an OD_600_ of 0.3 to 0.5. The staining of the bacterial membrane with Nile red was performed as described above. After Nile red staining, cells were fixed in 1.2% paraformaldehyde for 20 min at room temperature. Portions (1 to 1.5 µl) of the different samples were spotted on microscope slides as described above. Phase-contrast and fluorescence images were taken at a magnification of ×1,000 using a Zeiss Axioskop 40 microscope coupled to an AxioCam MRm camera and processed using the Axio Vision software (release 4.7). Nile red and mNeonGreen fluorescence signals were detected using the Zeiss filter sets 43 and 37, respectively.

### Transmission electron microscopy.

Overnight cultures of L. monocytogenes strains 10403S, 10403SΔ*eslB*_2_, and 10403SΔ*eslB*_2_ compl. were used to inoculate 25 ml BHI broth or BHI broth supplemented with 0.5 M sucrose to an OD_600_ of 0.05. Bacteria were grown at 37°C and 200 rpm for 3.5 h (BHI broth) or 6 h (BHI broth containing 0.5 M sucrose). A 15-ml portion of the cultures was centrifuged for 10 min at 4,000 rpm, and the cell pellet was washed twice in phosphate-buffered saline (127 mM NaCl, 2.7 mM KCl, 10 mM Na_2_HPO_4,_ 1.8 mM KH_2_PO_4_; pH 7.4) and fixed overnight in 2.5% (wt/vol) glutaraldehyde at 4°C. Cells were then mixed with 1.5% (wt/vol; final concentration in PBS) molten Bacto agar, which was kept liquid at 55°C. After solidification, the agar block was cut into pieces with a volume of 1 mm^3^. A dehydration series was performed (15% aqueous ethanol solution for 15 min, 30%, 50%, 70%, and 95% for 30 min, and 100% twice for 30 min) at 0°C, followed by an incubation step in 66% (vol/vol; in ethanol) LR white resin mixture (Plano) for 2 h at room temperature and embedded in 100% LR white solution overnight at 4°C. Next, the agar piece was transferred to a gelatin capsule filled with fresh LR white resin, which was subsequently polymerized at 55°C for 24 h. A milling tool (TM 60; Reichert & Jung, Vienna, Austria) was used to shape the gelatin capsule into a truncated pyramid. An ultramicrotome (Reichert Ultracut E; Leica Microsystems, Wetzlar, Germany) and a diamond knife (Delaware Diamond Knives, Wilmington, DE, USA) were used to obtain ultrathin sections (80 nm) of the samples. The resulting sections were mounted on mesh specimen grids (Plano) and stained with 4% (wt/vol) uranyl acetate solution (pH 7.0) for 10 min.

Microscopy was performed using a JEOL JEM 1011 transmission electron microscope (JEOL Germany GmbH, Munich, Germany) at 80 kV. Images were taken at a magnification of 30,000 and recorded with an Orius SC1000 charge-coupled device (CCD) camera (Gatan Inc., Pleasanton, CA). For each replicate, 20 cells were photographed and cell wall thickness was measured at three different locations using the ImageJ software (46). The average of the three measurements was calculated and the average and standard deviation of 20 cells plotted. The experiment was performed twice.

### Cell culture.

Bone marrow-derived macrophages (BMMs) were extracted from female C57BL/6 mice as described previously (47). BMMs were a gift from Charlotte S. C. Michaux and Sophie Helaine. BMMs were seeded at 5 × 10^5^ per well in a 24-well plate and grown overnight in 500 µl high-glucose Dulbecco’s modified Eagle medium (DMEM) at 37°C and 5% CO_2_. L. monocytogenes strains were grown overnight without shaking in 2 ml BHI medium at 30°C. The next morning, bacteria were opsonized with 8% mouse serum (Sigma-Aldrich) at room temperature for 20 min, and BMMs were infected for 1 h at a multiplicity of infection (MOI) of 2. BMMs were washed with PBS, and 1 ml DMEM containing 40 µg/ml gentamicin was added to kill extracellular bacteria. After 1 h, cells were washed with PBS and covered with 1 ml DMEM containing 10 µg/ml gentamicin. The number of recovered bacteria was determined 2, 4, 6, and 8 h postinfection. To this end, BMMs were lysed using 1 ml PBS containing 0.1% (vol/vol) Triton X-100, and serial dilutions were plated on BHI agar plates. The number of CFU was determined after incubation of the plates overnight at 37°C. Three technical repeats were performed for each experiment, and average values were calculated. Average values and standard deviations from three independent experiments were plotted.

### Drosophila melanogaster infections.

Fly injections were carried out with microinjection needles produced from borosilicate glass capillaries (TW100-4; World Precision Instruments) and a needle puller (model PC-10; Narishige). Injections were performed using a Picospritzer III system (Parker Hannifin), and the injection volume was calibrated by expelling a drop of liquid from the needle into a pot of mineral oil and halocarbon oil (both from Sigma). The expelled drop was measured using the microscope graticule to obtain a final injection volume of 50 nl. Flies were then anesthetized with CO_2_ and injected with either 50 nl of bacterial suspension in PBS or sterile PBS. Five- to seven-day-old age-matched male flies were used for all experiments. Flies were grouped into uninjected control flies, wounded control flies (injected with sterile PBS), and flies infected with L. monocytogenes. Each group consisted of 58 to 60 flies. All survival experiments were conducted at 29°C. Dead flies were counted daily. Food vials were placed horizontally to reduce the possibility of fly death from flies getting stuck to the food, and flies were transferred to fresh food every 3 to 4 days. For the quantification of the bacterial load, 16 flies per condition and per bacterial strain were collected at the indicated time points. The flies were homogenized in 100 µl of Tris-EDTA buffer (pH 8) containing 1% Triton X-100 and 1% proteinase K (P8107S; NEB). Homogenates were incubated for 3 h at 55°C followed by a 10-min incubation step at 95°C. Following incubation, qPCR was carried out using the *actA*-specific primers EGD-E_ActA_L1 and EGD-E_ActA_R1 to determine the number of bacterial CFU. PCR was performed with Sensimix SYBR green no-ROX (Bioline) on a Corbett Rotor-Gene 6000. The cycling conditions were as follows: hold at 95°C for 10 min, then 45 cycles of 95°C for 15 s, 57°C for 30 s, and 72°C for 30 s, followed by a melting curve. Gene abundances were calculated as previously described (48).

### Data availability.

The Illumina reads for the L. monocytogenes strains 10403SΔ*eslB*_1_, 10403SΔ*eslB*_2_, 10403SΔ*eslB*_1_ compl., and 10403SΔ*eslB*_2_ compl. were deposited in the European Nucleotide Archive under the accession number PRJEB40123.

## Supplementary Material

Supplemental file 1
