# Supplementary material for: EslB Is Required for Cell Wall Biosynthesis and Modification in Listeria monocytogenes
Source: J Bacteriol. 2021 Jan 25;203(4):e00553-20. doi: 10.1128/JB.00553-20 (PMC7847544; doi:10.1128/JB.00553-20)
Supplement: Supplemental file 1 [file JB.00553-20-s0001.pdf]

SUPPLEMENTAL MATERIAL FOR MANUSCRIPT:

EslB is required for cell wall biosynthesis and modification in *Listeria monocytogenes*

Jeanine Rismondo[a,b,#], Lisa M. Schulz[b], Maria Yacoub[a], Ashima Wadhawan[c], Michael Hoppert[b], Marc S. Dionne[c], Angelika Gründling[a,#]

[a] Section of Molecular Microbiology and Medical Research Council Centre for Molecular Bacteriology and Infection, Imperial College London, London SW7 2AZ.

[b] Department of General Microbiology, GZMB, Georg-August-University Göttingen, 37077 Göttingen, Germany

[c] Department of Life Sciences, Medical Research Council Centre for Molecular Bacteriology and Infection, Imperial College London, London SW7 2AZ.

[#]To whom correspondence should be addressed:

Jeanine Rismondo – jrismon@gwdg.de, Angelika Gründling – a.grundling@imperial.ac.uk

SUPPLEMENTAL TABLES

Table S1: Bacterial strains used in this study

Unique ID	Strain name and resistance	Source
***Escherichia coli* strains**		
ANG1264	DH5α pKSV7; AmpR	(1)
1265	XL1-Blue pKT25; KanR	(2)
1266	XL1-Blue pKNT25; KanR	(3)
1267	XL1-Blue pUT18; AmpR	(2)
1268	XL1-Blue pUT18C; AmpR	(2)
1269	XL1-Blue pKT25-*zip*; KanR	(4)
1270	XL1-Blue pUT18C-*zip*; AmpR	(4)
ANG4243	XL1-Blue pIMK3; KanR	(5)
ANG4236	XL1-Blue pKSV7-Δ*eslB*; AmpR	This study
ANG4647	XL1-Blue pIMK3-*eslB*; KanR	This study
ANG5660	XL1-Blue pPL3e-P_{eslA}-*eslABC*; CamR	This study
ANG5661	SM10 pPL3e-P_{eslA}-*eslABC*; KanR CamR	This study
EJR4	XL1-Blue pKNT25-*eslA*; KanR	This study
EJR5	XL1-Blue pKT25-*eslA*; KanR	This study
EJR6	XL1-Blue pUT18-*eslA*; AmpR	This study
EJR7	XL1-Blue pUT18C-*eslA*; AmpR	This study
EJR8	XL1-Blue pKNT25-*eslB*; KanR	This study
EJR9	XL1-Blue pKT25-*eslB*; KanR	This study
EJR10	XL1-Blue pUT18-*eslB*; AmpR	This study
EJR11	XL1-Blue pUT18C-*eslB*; AmpR	This study
EJR12	XL1-Blue pKNT25-*eslC*; KanR	This study
EJR13	XL1-Blue pKT25-*eslC*; KanR	This study
EJR14	CLG190 pUT18-*eslC*; AmpR	This study
EJR15	XL1-Blue pUT18C-*eslC*; AmpR	This study
EJR39	XL1-Blue pIMK2-*mNeonGreen-zapA*; KanR	This study
EJR43	XL1-Blue pKSV7-Δ*eslC*; AmpR	This study
EJR54	XL1-Blue pKSV7-Δ*eslA*; AmpR	This study
EJR60	S17-1 pIMK2-*mNeonGreen-zapA*; KanR	This study
***Listeria monocytogenes* strains**		
ANG1263	10403S; StrepR	(6)
ANG4275	10403SΔ$eslB_{(1)}$; StrepR	This study
ANG4678	10403S pIMK3-*eslB*; StrepR KanR	This study
ANG4688	10403SΔ$eslB_{(1)}$ pIMK3-*eslB* (or short: 10403SΔ$eslB_{(1)}$ compl.); StrepR KanR	This study
ANG5662	10403SΔ$eslB_{(2)}$; StrepR	This study
ANG5663	10403SΔ$eslB_{(2)}$ pPL3e-P_{eslA}-*eslABC* (or short: 10403SΔ$eslB_{(2)}$ compl.); ErmR StrepR	This study
LJR7	10403SΔ*eslC*; StrepR	This study
LJR21	10403SΔ*eslC* pPL3e-P_{eslA}-*eslABC* (or short: 10403SΔ*eslC* compl.); ErmR StrepR	This study
LJR28	10403S pIMK2-*mNeonGreen-zapA*; StrepR KanR	This study
LJR29	10403SΔ$eslB_{(2)}$ pIMK2-*mNeonGreen-zapA*; StrepR KanR	This study
LJR33	10403SΔ*eslA*; StrepR	This study
LJR34	10403SΔ*eslA* pPL3e-P_{eslA}-*eslABC* (or short: 10403SΔ*eslC* compl.); ErmR StrepR	This study

Table S2: Primers used in this study

Number	Name	Sequence
ANG2532	*eslB* up fw	GCGCGGATCCCCGCAATATGTGAAATTAGTATAATG
ANG2533	*eslB* up rev	TTGGAAAAGCCCTTCCCGGTACCATAACCCTCGATTAAACAT
ANG2534	*eslB* down fw	TGGTACCGGGAAGGGCTTTTCCAATTGTCTAAAACGAATTAA
ANG2535	*eslB* down rev	GCGCTCTAGAGCTCGCGCACTCTCATAAAC
ANG2812	pIMK3-*eslB* fw	GCGCCCATGGGGTTTAATCGAGGGTTATGGTACC
ANG2813	pIMK3-*eslB* rev	GCGCGTCGACTTAATTCGTTTTAGACAATTGGAAA
ANG3349	pPL3e-*eslABC* fw SalI	ACGCGTCGACCTGGATGTGGCGTAAGGG
ANG3350	pPL3e-*eslABC* rev BamHI	CGCGGATCCCATAACTTTGTCCCGATTGTCC
JR39	Neon_rev	GCCACTACTTGTCTTATAGAGTTCATCCATACCCA
JR40	Neon_ZapA_dw_f	GAACTCTATAAGACAAGTAGTGGCCTAAACGAATTT
JR44	B2H EslA fw XbaI	CTAGTCTAGAAAAAAATTCGGAACTTAACTAAAAAGATG
JR45	B2H EslA rev KpnI	CGGGGTACCGGAATCTCGTAATAATCTATTTTATCATTTG
JR46	B2H EslB fw XbaI	CTAGTCTAGAATTTAATCGAGGGTTATGGTACC
JR47	B2H EslB rev BamHI	CGCGGATCCCAATTCGTTTTAGACAATTGGAAAAGC
JR48	B2H EslC fw XbaI	CTAGTCTAGAAACGACAGAAACAGAAACGATTAAAC
JR49	B2H EslC rev KpnI	CGGGGTACCGGTAAGGATGGAATATTTTTCTTTTTCTTC
JR73	p3-mNeon-Zap fw NcoI	CATGCCATGGTTTCGAAAGGAGAGGAGGATAAT
JR74	p3-mNeon-Zap rev SalI	GCGCGTCGACTTAATCTCTTCCTTTAATTCGAGC
LMS155	*eslC* up fwd	CGCGGATCCAATTTTTCTTCGCCATCGCTTCC
LMS156	*eslC* down rev	CGGGGTACCCTGCATCATTACCATATAAACGGACG
LMS157	*eslC* down fwd	CTACATTAATATGCTTCGTTAAATTAGCTTTCAATTCAGC
LMS158	*eslC* up rev	ATTTAACGAAGCATATTAATGTAGCGTTAAGTCCGATTGTTAA
LMS159	*eslA* down rev	CCGGAATTCGCTTCTTTGTTTTTATACCCAGCATTAC
LMS160	*eslA* up fwd	CGCGGATCCGAGTACACTTAATATTACGCTAAAAATCACAG
LMS161	*eslA* up rev	AAAAGATGGACGCGATAGAAGACATTTTCACGGTGC
LMS162	*eslA* down fwd	TGTCTTCTATCGCGTCCATCTTTTTAGTTAAGTTCCGAA
	EGD-E_ActA_L1	AAGCGAAGGAAGAACCAGGG
	EGD-E_ActA_R1	CCCCTAAAGAGAACACGCCA

Table S3: Identified sequence alterations in *L. monocytogenes eslB* deletion and complementation strains.

Strain number	Reference position[1]	Type[2]	Ref[3]	Allele[4]	Frequency[5]	Average Quality[6]	Annotations	AA change[7]
ANG4275 10403SΔ*eslB*$_{(1)}$	2425786-2425875	DEL			100%		*lmo2396*, internalin	30 aa deletion
ANG4688 10403SΔ*eslB*$_{(1)}$ compl.	2366318	SNV	C	A	100%	37.04	*lmo2342*, 16S pseudo uridylate synthase	Val186Leu
ANG5662 10403SΔ*eslB*$_{(2)}$	–							
ANG5663 10403SΔ*eslB*$_{(2)}$ compl.	2058478	DEL	A	–	100%	36.33	*lmo2022,* NifS-like protein required for NAD biosynthesis	His171fs

[1] Reference position is based on the position in the *L. monocytogenes* 10403S reference genome (NC_01744).

[2] Type of mutation: SNV = single nucleotide variant; DEL = nucleotide deletion.

[3] Ref indicates base in reference genome.

[4] Allele indicates base at the same position in the sequenced strain.

[5] Frequency at which the base change was found in the sequenced strain.

[6] Average quality score.

[7] AA change indicates the resulting amino acid change in the protein found in the reference strains as compared to the sequenced strain.

SUPPLEMENTAL FIGURES

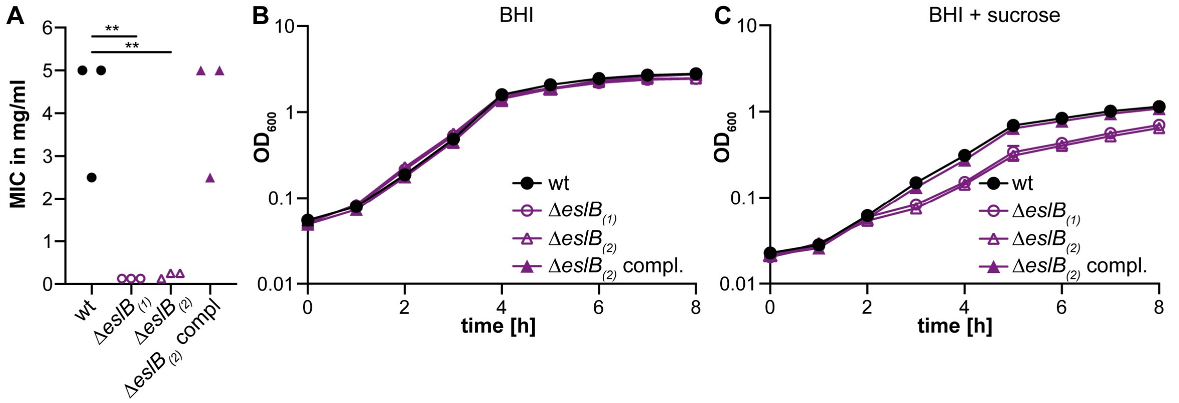

Figure S1: Comparison of lysozyme sensitivity and growth of two different *eslB* mutants and a complementation strain. (A) The minimal inhibitory concentration for lysozyme was determined for *L. monocytogenes* strains 10403S (wt), 10403SΔ*eslB*$_{(1)}$, 10403SΔ*eslB*$_{(2)}$ and 10403SΔ*eslB*$_{(2)}$ compl. using a microbroth dilution assay. The results of three independent experiments are shown. For statistical analysis, a one-way ANOVA followed by a Dunnett's multiple comparisons test was used (** $p \leq 0.01$). (B-C) Growth analysis of *L. monocytogenes* strains 10403S (wt), 10403SΔ*eslB*$_{(1)}$, 10403SΔ*eslB*$_{(2)}$ and 10403SΔ*eslB*$_{(2)}$ compl. in (B) BHI broth or (C) BHI broth containing 0.5 M sucrose. All cultures were incubated at 37°C and the bacterial growth monitored by determining OD$_{600}$ readings at hourly intervals. The average OD$_{600}$ readings and standard deviations were calculated from three independent experiments and plotted.

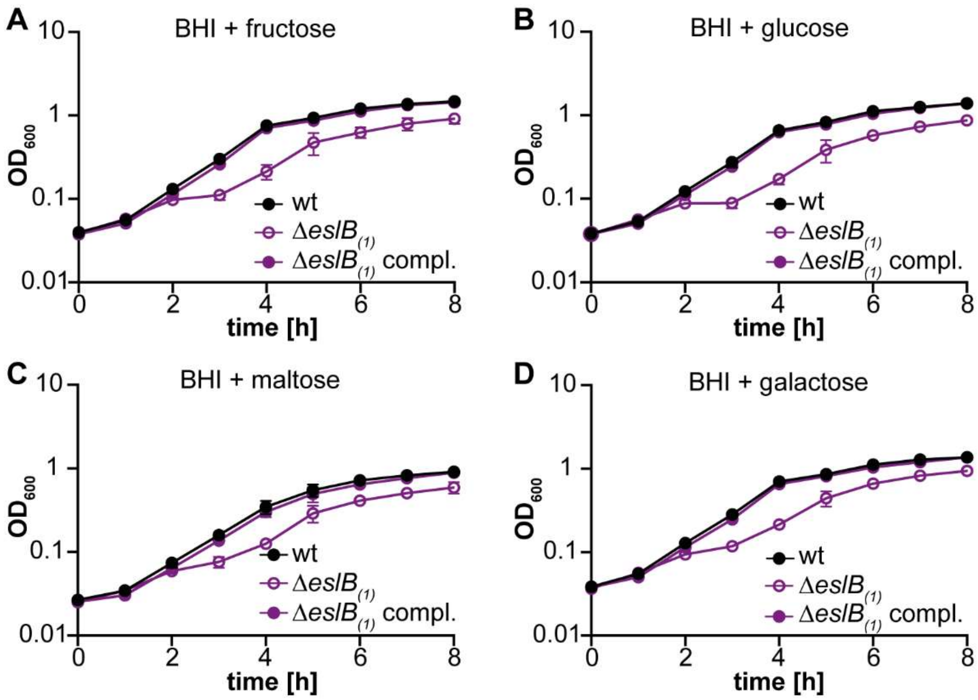

Figure S2: The *L. monocytogenes eslB* mutant has a growth defect in BHI medium containing 0.5 M added sugar. *L. monocytogenes* strains 10403S (wt), 10403SΔ*eslB(1)* and the complementation strain 10403SΔ*eslB(1)* compl. were grown in BHI broth containing (A) 0.5 M fructose, (B) 0.5 M glucose, (C) 0.5 M maltose or (D) 0.5 M galactose. Strain 10403SΔ*eslB(1)* compl. was grown in the presence of 1 mM IPTG. All cultures were incubated at 37°C and bacterial growth was monitored by determining OD_{600} readings at hourly intervals. Average OD_{600} values and standard deviations were calculated from three independent experiments and plotted. Of note, some of the error bars are too small to be seen on the graph.

REFERENCES

1. Smith K, Youngman P. 1992. Use of a new integrational vector to investigate compartment-specific expression of the *Bacillus subtilis spoIIM* gene. Biochimie 74:705-11.
2. Karimova G, Ullmann A, Ladant D. 2001. Protein-protein interaction between *Bacillus stearothermophilus* tyrosyl-tRNA synthetase subdomains revealed by a bacterial two-hybrid system. J Mol Microbiol Biotechnol 3:73-82.
3. Karimova G, Dautin N, Ladant D. 2005. Interaction network among *Escherichia coli* membrane proteins involved in cell division as revealed by bacterial two-hybrid analysis. J Bacteriol 187:2233-43.
4. Karimova G, Pidoux J, Ullmann A, Ladant D. 1998. A bacterial two-hybrid system based on a reconstituted signal transduction pathway. Proc Natl Acad Sci U S A 95:5752-6.
5. Monk IR, Gahan CG, Hill C. 2008. Tools for functional postgenomic analysis of *Listeria monocytogenes*. Appl Environ Microbiol 74:3921-34.
6. Bishop DK, Hinrichs DJ. 1987. Adoptive transfer of immunity to *Listeria monocytogenes*. The influence of in vitro stimulation on lymphocyte subset requirements. J Immunol 139:2005-9.
